# Amyloid A and lactic acid as a predictor in patients with sepsis in patients with liver cirrhosis

**DOI:** 10.1186/s12876-024-03326-4

**Published:** 2024-07-22

**Authors:** Qiang Li, Rui Zeng, Yunxia Sun, Weipeng Xu, Zhihua Xie, Bencai Jing, Ting Zhang

**Affiliations:** 1 Emergency Medicine, Fushun People’s Hospital, Zigong, Sichuan China; 2 Department of Critical Care Medicine, Fushun People’s Hospital, Zigong, Sichuan China

**Keywords:** Liver cirrhosis, Sepsis, SAA, BLA

## Abstract

**Background:**

Sepsis is triggered by pathogenic microorganisms, resulting in a systemic inflammatory response. Liver cirrhosis and sepsis create a vicious cycle: cirrhosis weakens immune function, raising infection risk and hindering pathogen clearance. Optimal treatment outcomes depend on understanding liver cirrhosis patients’ sepsis risk factors. Thus, preventing sepsis involves addressing these risk factors. Therefore, early identification and understanding of clinical characteristics in liver cirrhosis patients with sepsis are crucial for selecting appropriate antibiotics. A case-control study using logistic regression was conducted to examine the prognostic value of amyloid A/lactate level monitoring in identifying sepsis risk factors in liver cirrhosis patients.

**Methods:**

From March 2020 to March 2022, 136 liver cirrhosis patients treated at our hospital were divided into a sepsis group (*n* = 35) and a non-sepsis group (*n* = 101) based on sepsis complications. General clinical data were collected. Univariate analysis screened for liver cirrhosis patients’ sepsis risk factors. Multivariate logistic analysis was subsequently employed to evaluate the risk factors. Sepsis patients were followed up for a month. Based on prognosis, patients were categorized into a poor prognosis group (*n* = 16) and a good prognosis group (*n* = 19). Serum amyloid A (SAA) and blood lactic acid (BLA) levels were compared between the two groups. The receiver operating characteristic (ROC) curve was used to evaluate the prognostic value of both individual and combined SAA/BLA monitoring.

**Results:**

Patient data, including age, diabetes history, liver cancer, hepatic artery embolization, recent antibiotic use, invasive procedures within two weeks, APACHE II Scoring, ALB and SAA and BLA levels, were compared between the sepsis and non-sepsis groups, showing significant differences (*P* < 0.05). Logistic regression identified factors such as age ≥ 70, recent antibiotic use, recent invasive procedures, history of liver cancer, hepatic artery embolization history, high APACHE II scores, decreased albumin, and elevated SAA and BLA levels as independent sepsis risk factors in liver cirrhosis patients (*P* < 0.05). Among the 35 sepsis patients, 16 had a poor prognosis, representing an incidence rate of 45.71%. Serum SAA and BLA levels were significantly higher in the poor prognosis group than in the good prognosis group (*P* < 0.05). The AUC for serum SAA and BLA was 0.831 (95%CI: 0.738–0.924), 0.720 (95%CI: 0.600–0.840), and 0.909 (95%CI: 0.847–0.972), respectively. The combined diagnostic AUC was significantly higher than that of single factor predictions (*P* < 0.05). The predictive value ranked as follows: joint detection > SAA > BLA.

**Conclusion:**

In treating liver cirrhosis, prioritize patients with advanced age, a history of hepatic artery embolization, recent invasive operations, history of liver cancer, recent antibiotic exposure, high APACHE II scores and low albumin. Closely monitoring serum SAA and BLA levels in these patients can offer valuable insights for early clinical prevention and treatment.

## Introduction

Sepsis is a systemic inflammatory response triggered by infection with pathogenic microorganisms. This complex immune response is triggered by microbial antigens and mediated by cells and cytokines [1, 2]. Immune disorders can cause cell damage and microcirculation disorders, leading to severe sepsis, septic shock, and multiple organ dysfunction syndrome (MODS) [3, 4]. Sepsis is characterized by inflammatory responses and coagulation dysfunction, leading to insufficient organ and tissue perfusion, potentially resulting in MODS and death. Sepsis mortality rates range from 30–70%. Recent studies indicate MODS as the primary cause of death in sepsis patients, responsible for 43.1% of deaths [5, 6]. Evidence increasingly shows that sepsis survivors often face long-term physical, psychological, and cognitive impairments, affecting their health care and community life [7].

The incidence of sepsis in liver disease patients is rising in both Britain and the United States, with sepsis causing death in up to 55% of liver cirrhosis cases [8, 9]. A vicious cycle exists between liver cirrhosis and sepsis: cirrhosis diminishes the body’s immune function, increasing infection risk and reducing pathogen clearance. Conversely, systemic immune inflammatory reactions from infections further deteriorate liver function, exacerbating hepatic encephalopathy, acute renal failure, and upper gastrointestinal bleeding [10]. Consequently, liver cirrhosis elevates the risk of sepsis, along with sepsis-induced organ failure and mortality [11].

Therefore, early diagnosis of sepsis in liver cirrhosis patients is crucial for timely interventions that can reduce mortality rates. However, currently, there is no ideal diagnostic method for sepsis. Serum amyloid A (SAA) is an acute-phase protein in plasma involved in immune response, lipid metabolism, and inflammatory defense, widely used as a clinical marker of inflammation [12]. Additionally, studies have indicated that blood lactic acid (BLA) levels serve as a critical marker for assessing tissue hypoperfusion in sepsis patients [13]. Therefore, we explored the efficacy of serum SAA and BLA measurements for the early diagnosis of sepsis in liver cirrhosis patients. To identify early clinical characteristics of liver cirrhosis, we studied 136 patients with liver cirrhosis and sepsis admitted to our hospital between March 2020 and March 2022, aiming to identify sepsis risk factors and provide a clinical basis for its prevention in these patients.

## Patients and methods

**Fig. 1:**
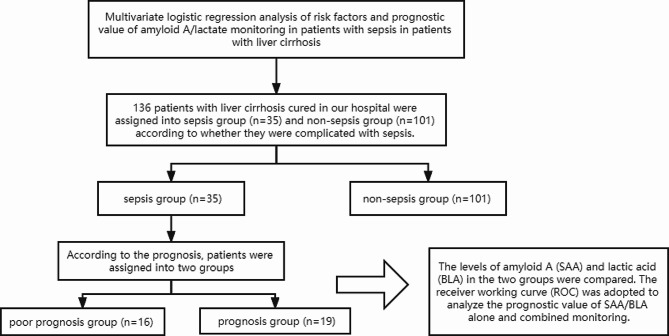
Article flow chart

From March 2020 to March 2022, 136 liver cirrhosis patients treated in our hospital were divided into a sepsis group (*n* = 35) and a non-sepsis group (*n* = 101), based on the presence of sepsis complications. General data for both groups is presented in Table 1. The study was reviewed and approved by our hospital’s Medical Ethics Review Committee. Sepsis patients were followed for one month. Based on prognosis, patients were categorized into a poor prognosis group (*n* = 16) and a good prognosis group (*n* = 19). Prognostic criteria: Patients with septic shock were divided into good and poor prognosis groups based on outcomes. Good prognosis criteria: Noticeable improvement or normalization of tissue perfusion, inflammation, and organ function after one month of treatment. Poor prognosis criteria: Lack of improvement in the aforementioned indexes or patient death during treatment. The study’s flow chart is provided in Fig. 1.

Inclusion criteria: (1) All cases were diagnosed with liver cirrhosis based on medical history, symptoms, physical signs, biochemical tests, imaging studies, or liver histopathology, with diagnostic criteria derived from relevant literature [14]. Patients with malignant tumors, diabetes, HIV infection, hormone therapy, and use of drugs affecting immune function were excluded. (2) Diagnostic criteria for liver cirrhosis complicated by sepsis are based on cirrhosis diagnosis and reference to the 2018 Chinese guidelines for treating severe sepsis/septic shock [15]: (1) Confirmation of bacterial presence or highly suspected infection sites; (2) Other indicators matching the systemic inflammatory response syndrome (SIRS) diagnostic criteria [16], treated with antibiotics; (3) Inclusion criteria for the non-sepsis group: liver cirrhosis patients without any infection and with complete data; (4) Patients signed the informed consent form for the study.

Exclusion criteria: (1) Patients with lung cancer, gastric cancer, and other systemic malignant tumors confirmed by CT, MRI, or histopathology; (2) Patients with a history of liver transplantation; (3) Patie nts who had not received antibiotic treatment; (4) Patients with severe mental illness who could not communicate normally; (5) Patients who had participated in similar research studies.

### Treatment methods

#### Laboratory examination

On admission day, fasting venous blood samples from patients were collected. The hospital’s laboratory department performed routine blood tests. Amyloid A (SAA) levels in all subjects were measured using an enzyme-linked immunosorbent assay (ELISA). Procedures followed the SAA detection kit instructions by Qiyi Biotechnology (Shanghai) Co., Ltd. Blood lactic acid levels (BLA) were detected using the Danish Ledu ABL825FLEX automatic blood gas analyzer. If infection occurred, blood samples were promptly collected at the time of infection, and the aforementioned tests were conducted by our hospital’s laboratory.

### Observation index

Upon patient admission, the following data were recorded: (a) General information: age, gender, liver cirrhosis etiology, and history of hepatic artery embolization; (b) Comorbidities: hypertension, diabetes, liver cancer; c. Laboratory indicators: blood routine, Serum creatinine, albumin, Acute Physiology and Chronic Health Evaluation II (APACHE II), among others; d. Recent medical history: antibiotic exposure within 30 days, invasive procedures within the last 2 weeks, proton pump inhibitor use, liver hardness, and Child-Turcotte-Pugh (CTP) classification.

### Statistical analysis

Data analysis was performed using SPSS 22.0. Measurement data fitting a normal distribution and homogeneous variance were expressed as mean ± standard deviation (x ± s). For comparison, independent samples t-test was used; for non-normal distributions, median (interquartile range) [M (P25, P75)] was used, employing the rank sum test. Enumeration data were reported as percentages and case numbers, with the χ2 test applied. Binary logistic regression analysis was used to identify influencing factors of sepsis in liver cirrhosis patients. The receiver operating characteristic (ROC) curve was employed to evaluate the combined prognostic value of amyloid A and lactic acid level monitoring in sepsis patients. A *P*-value < 0.05 was considered statistically significant.

## Results

### Comparison of the general situation

This study included 136 liver cirrhosis patients, comprising 35 with sepsis and 101 without sepsis. Among the 35 sepsis patients with liver cirrhosis, the average age was 72.31 ± 8.64 years, with 23 males and 12 females. Male incidence was higher than female. There was no statistically significant difference (*P* > 0.05) in gender, history of hypertension, use of other drugs, use of proton pump inhibitors, liver stiffness, CTP grade, between the sepsis and non-sepsis groups. Ignificant differences were observed in terms of age, history of diabetes, liver cancer, history of hepatic artery embolization, antibiotic exposure within 30 days, history of invasive procedures in the past 2 weeks and APACHE II Scoring (*P* < 0.05). All results can be found in Table 1 .

**Table 1:** The general situation

Variable	Sepsis group(*n* = 35)	Non-sepsis group (*n* = 101)	t/X2	*P*
Age (years)	72.31 ± 8.64	65.42 ± 9.07	3.919	<0.05
Gender (male / female)	23/12	58/43	0.741	>0.05
high blood pressure	18(51.43)	41(40.59)	1.242	>0.05
diabetes	30(85.71)	21(20.79)	46.744	<0.05
Liver cancer	20(57.14)	8(7.92)	38.518	<0.05
History of hepatic artery embolization	27(77.14)	10(9.90)	59.343	<0.05
Antibiotic exposure within 30 days	32(91.43)	21(20.79)	54.530	<0.05
Use of other drugs				
diuretics	8(22.85)	28(27.72)	0.823	>0.05
lactulose	25(71.42)	80(79.20)	1.525	>0.05
History of invasive operation in the past 2 weeks	17(48.57)	5(4.95)	36.475	<0.05
Application of proton pump inhibitor	18(51.43)	54(53.47)	0.043	>0.05
Liver hardness(kPa)	31.30 ± 5.16	30.66 ± 5.27	0.622	>0.05
CTP Grading			3.112	>0.05
B	10(28.57)	36(35.64)		
C	25(71.42)	65(64.36)		
APACHE II Scoring (points)	25.37 ± 9.01	18.04 ± 6.34	5.254	<0.05

### Laboratory index comparison

No significant differences were observed in WBC, ALT and Serum creatinine levels between the two groups (*P* > 0.05). Significant differences were found in ALB, SAA and BLA levels, with the sepsis group showing higher values than the non-sepsis group (*P* < 0.05). The results are detailed in Table 2.

**Table 2:** Laboratory index comparison

Variable	WBC( × 109/L)	ALT(U/L)	Scr(µmol/L)	ALB (g/L)	SAA (µmol/L)	BLA (mmol/L)
Non-sepsis group(*n* = 101)	8.31 ± 2.13	76.31 ± 11.03	106.1 ± 15.6	30.36 ± 4.76	7.38 ± 2.19	1.04 ± 0.62
Sepsis group(*n* = 35)	8.27 ± 5.44	77.28 ± 12.87	115.6 ± 16.9	25.85 ± 5.65	128.34 ± 41.37	5. 14 ± 1.03
T	0.062	0.429	0.582	6.716	29.472	28.031
*P*	> 0.05	> 0.05	> 0.05	<0.05	<0.05	<0.05

### Analysis of multifactorial influences on sepsis in liver cirrhosis patients

We used the statistically significant factors from univariate analysis as independent variables in a multivariate logistic regression model to identify the influencing factors. The detailed assignment table is presented in Table 3. The results showed that factors like age ≥ 70 years, history of liver cancer, antibiotic exposure within 30 days, recent history of invasive procedures, hepatic artery embolization, decreased albumin, high APACHE II scores, elevated serum SAA and BLA levels significantly influenced sepsis occurrence in liver cirrhosis patients (*P* < 0.05). All results are detailed in Table 4.

**Table 3:** Analysis of influencing factors of sepsis in patients with liver cirrhosis

Related factors	Variable name	Variable assignment
Age	X1	1 ≥ 70,2<70
diabetes	X2	1 = Yes,0 = None
Liver cancer	X3	1 = Yes,0 = None
History of hepatic artery embolization	X4	1 = Yes,0 = None
Antibiotic exposure within 30 days	X5	1 = Yes,0 = None
History of invasive operation in the past 2 weeks	X6	1 = Yes,0 = None
APACHE II Scoring	X_7_	1 = Too high,0 = Normal / low
ALB (g/L)	X_8_	1 = Too high,0 = Normal / low
SAA(µmol/L)	X_9_	1 = Too high,0 = Normal / low
BLA(mmol/L)	X_10_	1 = Too high,0 = Normal / low

**Table 4:** Logistics regression analysis of factors related to sepsis in patients with liver cirrhosis

Variable	Β	S.E.	Waldx2	*P* Value	OR Value(95%CI)	
Age	1.411	0.323	19.083	0.000	4.100	(2.177–7.722)
Diabetes	0.392	0. 114	11.824	0.157	1.480	(1.184–1.850)
Liver cancer	0.613	0.051	144.471	0.000	1.846	(1.670–2.040)
History of hepatic artery embolization	1.003	0.181	30.708	0.000	2.726	(1.912–3.887)
Antibiotic exposure within 30 days	2.313	1.020	5.142	0.023	10.105	(1.369–74.604)
History of invasive operation in the past 2 weeks	1.082	0.322	11.291	0.001	2.951	(1.570–5.546)
APACHE II Scoring	1.045	0.182	32.968	0.000	2.834	(1.990–4.062)
ALB (g/L)	0.357	0.422	4.325	0.000	1.829	(1.240–2.928)
SAA(µmol/L)	0.887	0.371	5.716	0.017	2.428	(1.173–5.024)
BLA(mmol/L)	0.603	0.215	7.866	0.005	1.828	(1.199–2.785)

### Liver cancer, serum SAA and ALT levels between good prognosis group and poor prognosis group

**Fig. 2:**
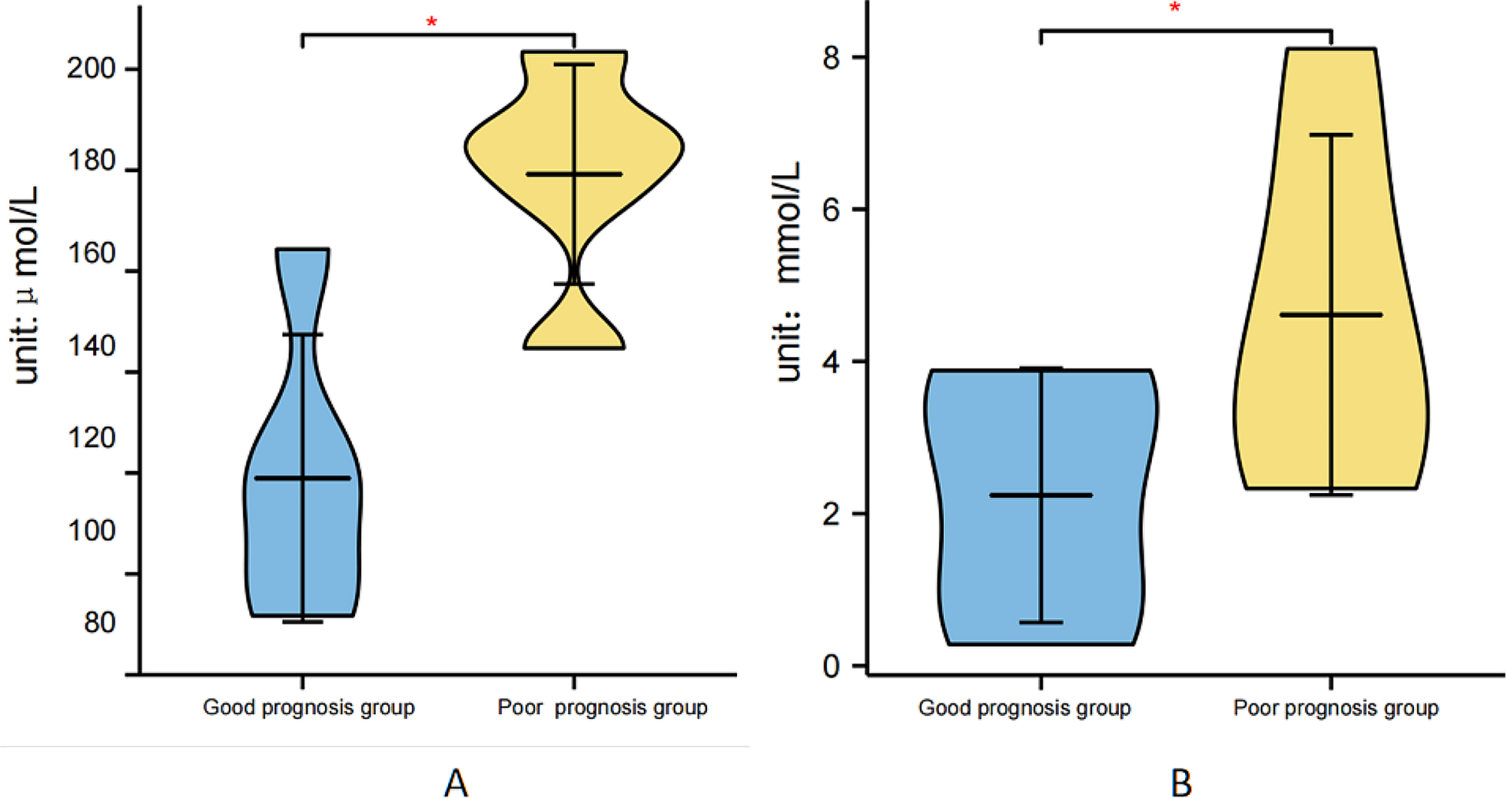
The serum SAA and BLA levels between good prognosis group and poor prognosis group. Note: **A:** Serum SAA level; **B:** Serum BLA level, comparison between groups, *P < 0.05

Among the 35 sepsis patients, 16 had a poor prognosis, representing an incidence rate of 45.71%. The comparison of laboratory indices revealed significantly higher serum SAA and BLA levels in the poor prognosis group compared to the good prognosis group (*P* < 0.05). All results are detailed in Fig. 2. There were 12 and 8 patients with liver cancer in the poor prognosis group and good prognosis, respectively, and there was no significant difference between the two groups (*P*<0.05).

### Prognostic value of single and combined detection of serum SAA and BLA in patients with sepsis

**Fig. 3:**
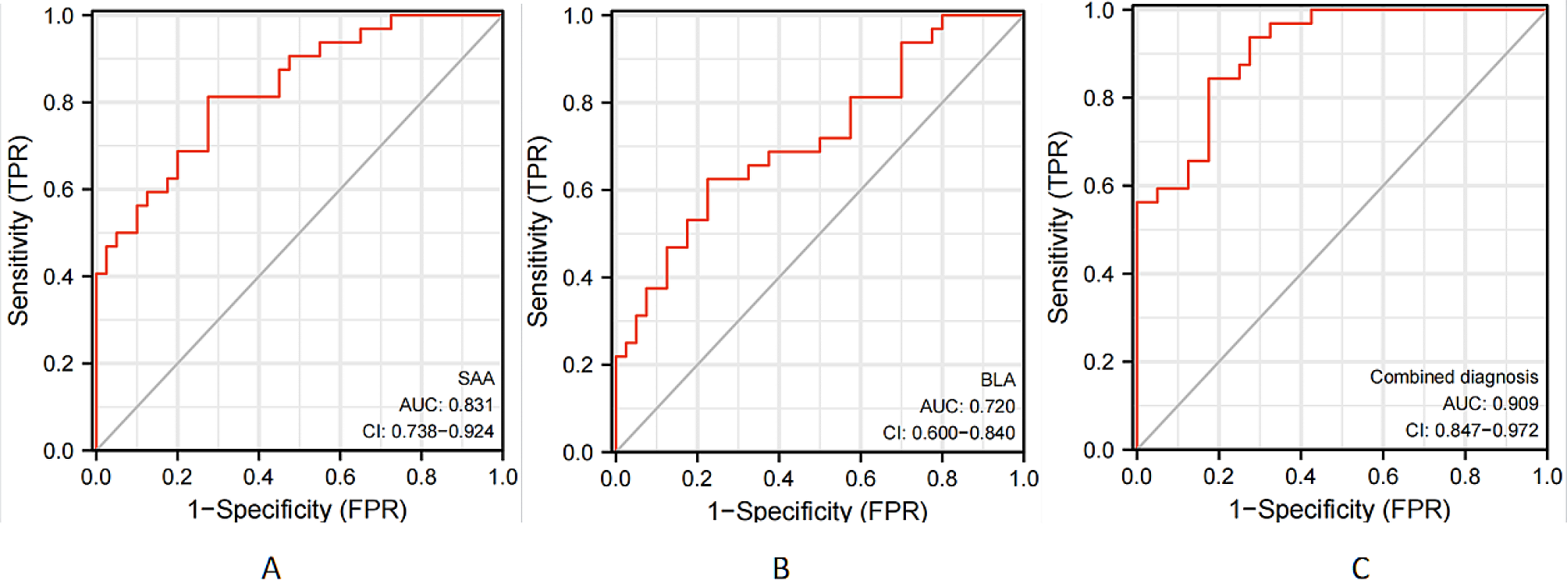
ROC curve for predicting the prognosis of patients with sepsis by single and combined detection of serum SAA and BLA. Note: **A:** Serum SAA level; **B:** Serum BLA level; **C:** Combined detection

Among the 35 sepsis patients, 16 had a poor prognosis, with an incidence rate of 45.71%. Serum SAA and BLA levels were significantly higher in the poor prognosis group compared to the good prognosis group, based on laboratory index levels (*P* < 0.05). All results are detailed in Fig. 2. ROC curve analysis showed serum SAA and BLA AUCs to be 0.831 (95%CI: 0.738–0.924), 0.720 (95%CI: 0.600–0.840), and 0.909 (95%CI: 0.847–0.972), respectively. Compared to single factor prediction, the combined diagnostic AUC was significantly higher (*P* < 0.05). Predictive value ranked as follows: combined detection > SAA > BLA, as shown in Fig. 3; Table 5.

**Table 5:** Prognostic efficacy of single and combined detection of serum SAA and BLA in patients with sepsis

Variable	AUC	*P* value	Cut-off Value	sensitivity(%)	Specificity degree(%)	Yoden index	95%CI
SAA	0.831	0.027	132.47	81.25	72.53	0.538	0.738–0.924
BLA	0.720	0.038	2.093	77.53	69.08	0.400	0.600–0.840
Joint diagnosis	0.909	0.015	1.426	84.42	82.56	0.669	0.847–0.972

## Discussion

Liver cirrhosis patients commonly develop bacterial infections and sepsis, both life-threatening complications. Over the past few decades, treatments for liver cirrhosis, including antiviral therapy, portal hypertension management, and liver transplantation, have seen improvements. Despite these improvements, infection rates and mortality remain high in liver cirrhosis patients, particularly in those with decompensated cirrhosis, without substantial changes [17]. The causes of bacterial infections in cirrhosis are complex, involving factors like host immunity, pathogens, and liver and other organ functions, with immune disorders and micro-ecological changes in cirrhotic patients being particularly significant [18, 19]. Infections can cause dysfunctional haemodynamics and an over-reaction of inflammatory cytokines in cirrhotic patients, leading to serious complications like shock, liver failure, renal failure, and death. Sepsis in cirrhosis patients is a leading cause of ICU admissions and mortality, according to statistics. Reports of multi-drug resistant bacteria are increasing annually across various regions, making the recommended empirical antibiotic therapy often ineffective against hospital and healthcare-associated infections. hus, addressing bacterial infections in cirrhosis requires early intervention for risk factors, clinical characterization of infection types, and effective antibiotic therapy as critical issues.

This study included 136 liver cirrhosis patients, comprising 35 with sepsis and 101 without. Among the 35 sepsis patients with liver cirrhosis, the average age was 72.31 ± 8.64 years, including 23 males and 12 females. Male incidence was higher than female. Significant differences were observed in age, diabetes history, history of liver cancer, history of hepatic artery embolization, recent antibiotic exposure, invasive procedures in the last 2 weeks, APACHE II Scoring, and ALB, SAA and BLA levels. Logistic regression analysis revealed that factors such as age ≥ 70, recent antibiotic exposure, history of invasive operations in the past 2 weeks, history of liver cancer, hepatic artery embolization, decreased albumin, high APACHE II scores, and elevated SAA and BLA levels were all significantly associated. Liver cirrhosis patients are at an increased risk of sepsis due to these conditions. Elderly patients often have chronic conditions like hypertension and diabetes, which increase hospitalization rates, extend hospital stays, and raise the risk of infection after pathogen exposure. Most patients with liver cancer have a history of hepatitis or cirrhosis, have varying degrees of damage to liver function, often have low immunity, and have an increased risk of infection. Hepatic artery embolization, being an invasive procedure, indirectly raises the infection risk in patients [20–23]. Theoretically, invasive operations increase the risk of bacterial invasion and infection. As invasive operations increase, so does the probability of sepsis. Therefore, indications for invasive procedures should be carefully evaluated and strictly followed, with aseptic techniques implemented to minimize infection risk in clinical settings. Hypoalbuminemia is associated with the acquisition and severity of viral, bacterial, and fungal infections and predicts infectious complications of noninfectious diseases. Hypoalbuminemia is often due to inflammationbut can also be caused by hepatocyte damage and decreased albumin synthesis, dietaryinsufficiency of amino acids, or increased excretion of albumin. In addition, recent studies have shown that increased inflammatory markers and interleukin-6 concentrations in serum and ascites appear to be associated with severe hypoalbuminemia. Supplementation with functional albumin molecules has been shown to be beneficial for infection control in patients with cirrhosis. A prospective study found that high-dose albumin infusion reduced plasma cytokine levels and significantly reduced systemic inflammatory responses in patients with cirrhosis. Thus, albumin attenuates inflammatory injury by regulating plasma osmolality, improving microcirculation, increasing mesenteric blood flow, and reducing leukocyte rolling and adhesion. In addition, low serum albumin leels were a risk factor for liver cirrhosis patients with sepsis, correction of low serum albumin leels may improve the prognosis of patients with this kind of ending. However, in our study, it was found that 20 of 35 patients with sepsis had cancer, of which 12 (75.0%) patients with liver cancer in the poor prognosis group were significantly higher than 8 (42.1%) patients with liver cancer in the good prognosis group. May be related to cancer of the liver patient’s own accelerate protein decomposition, synthesis, exogenous albumin supplementation does not effectively maintain blood albumin levels, so in this case the input albumin to improve the prognosis of confirmed that need to be studied further. The synthesis of albumin constitutes about 50% of the synthesized proteins in the liver, liver cancer impairs the function of liver to synthesize albumin, resulting in a decrease in albumin level. In addition, liver cancer can damage liver tissue, leading to a decrease in its ability to clear pathogens and detoxify, increasing the risk of infection. Second, patients with liver cancer often have portal hypertension, which causes gastrointestinal congestion, bacterial translocation, and causes bacteremia. Meanwhile, portal hypertension leads to hypoalbuminemia, which increases the risk of spontaneous peritonitis and pulmonary infection [24–27]. The APACHE II scoring system, a comprehensive measure, is commonly used for critically ill patients [28]. It effectively assesses patients’ pathological states. A higher score is associated with a worse prognosis. In this study, sepsis patients had higher APACHE II scores compared to those without sepsis. This finding suggests that the APACHE II score is significantly valuable for the early clinical diagnosis of sepsis. Recent antibiotic exposure (within 30 days) significantly increases the risk of multidrug-resistant bacteria, leading to prolonged illness in infected patients, alig ning with prior research [29]. SAA, an acute phase reactive protein in plasma, serves as a common clinical marker for inflammation, participating in immune responses, lipid metabolism, and inflammatory defense [12]. In healthy individuals, SAA levels are low; however, infection by bacteria or viruses causes cytokines such as interleukin-1, interleukin-6, and tumor necrosis factor to prompt the liver to secrete SAA, leading to a significant increase in its levels within 8 to 12 h. SAA levels can peak, reaching up to 1000 times the normal value in severe cases [30]. SAA levels rise quickly following infection, making dynamic monitoring of SAA crucial for early septic shock diagnosis. Studies indicate SAA plays a role in the development of tumors and autoimmune diseases by mediating various signals during the body’s inflammatory response. Li Shouwei’s research found SAA levels in shock patients to be significantly higher compared to those in patients with less severe infections [31]. This study observed a significant increase in serum SAA levels in septic shock patients, indicating an association with septic shock’s development and pathogenesis. Furthermore, SAA is anticipated to serve as both a monitoring index and therapeutic target for septic shock’s progression. Thus, enhancing SAA level monitoring in cirrhosis patients is crucial for preventing sepsis following a sharp increase in serum SAA. Clinically, focusing on the treatment and care of cirrhosis patients with high SAA levels is essential to minimize sepsis incidence. Finally, sepsis patients experience multiple tissue microcirculation disorders, cell metabolism imbalance, hypoxia-ischemia, and anaerobic metabolism due to oxygen deficiency. Under sufficient oxygen, pyruvate is oxidized to enter the tricarboxylic acid cycle. In cases of tissue hypoxia or insufficient perfusion, pyruvate is converted to lactate first. Hence, a blood lactate level > 2.0 mmol/L is commonly used as a clinical indicator of tissue hypoperfusion in sepsis patients [13]. Serum creatinine has been shown to be an important predictor of sepsis, and it increases with age [32]. However, in this study, although the age was significantly higher in the sepsis group than in the non-sepsis group, there was no significant difference in serum creatinine levels between them, which may be related to the increase in serum creatinine due to the use of more diuretics in the non-sepsis group.

This study followed cirrhosis patients with sepsis for one month. The results showed that of the 35 sepsis patients, 16 had a poor prognosis, representing a 45.71% incidence rate. Serum SAA and BLA levels were significantly higher in the poor prognosis group than in the good prognosis group. Evidence suggests that SAA and BLA levels can predict sepsis patients’ prognosis. This study used the ROC curve to analyze the predictive power of SAA, BLA, and their combined monitoring. The results showed that the AUCs for serum SAA and BLA detection alone, and their combined detection were 0.831, 0.720, and 0.909, respectively. Compared to single-factor predictions, the combined diagnosis showed a significantly higher AUC. This indicates that SAA and BLA could serve as effective reference indicators for clinically predicting sepsis prognosis, with combined monitoring offering greater value. Therefore, monitoring CRP and PCT levels in sepsis patients should be emphasized. Understanding changes in their condition and timely adjusting treatment plans will help improve patient prognosis. Despite its strengths, this study has limitations to consider. For instance, the detected serum indices represented only specific timepoints. he study’s small and regional sample size necessitates expansion to ensure result accuracy.

In summary, sepsis can significantly worsen the clinical progression of liver cirrhosis. Age ≥ 70 years, recent antibiotic use, history of invasive operations within the last 2 weeks, hepatic artery embolization, decreased albumin, high APACHE II scores and elevated serum SAA and BLA levels are independent risk factors for sepsis in liver cirrhosis patients. Combined monitoring of SAA and BLA levels aids in the early diagnosis of sepsis in liver cirrhosis patients, enabling timely and appropriate treatment. This is crucial for preventing severe liver disease and saving lives.

## Data Availability

The datasets used and/or analysed during the current study are available from the corresponding author on reasonable request.
